# Impact of self-expandable metallic prosthesis on lymph node dissemination in obstructive left-sided colorectal cancer

**DOI:** 10.1007/s00464-025-11652-1

**Published:** 2025-04-14

**Authors:** Luciano Delgado-Plasencia, Antonio Boluda-Aparicio, Patricia Marrero-Marrero, Eduardo Salido-Ruíz, Esther Torres-Monzón, Carmen Peñalver-Alcaraz, Caroline Phillbrick, Alejandro Jiménez-Sosa

**Affiliations:** 1 University of La Laguna, San Cristóbal de La Laguna, Spain; 2 Department of General and Digestive Surgery, Hospital Universitario de Canarias, San Cristóbal de La Laguna, Spain; 3 Department of Pathology, Hospital Universitario de Canarias. La Laguna, Tenerife, Spain; 4 Nursing Department, Hospital Universitario de Canarias, La Laguna, Tenerife, Spain; 5 Department of Radiation Oncology, Hospital Universitario de Canarias, La Laguna, Tenerife, Spain; 6 Department of Research Unit, Hospital Universitario de Canarias, La Laguna, Tenerife, Spain

**Keywords:** Self-expandable metallic stent, Lymphatic vessels, Malignant colorectal obstruction

## Abstract

**Background:**

The introduction of self-expandable metal stent (SEMS) insertion in obstructive colorectal cancer (CRC) has been associated with an increased risk of tumor perforation, potentially leading to peritoneal dissemination, tumor cell spread via lymphatic vessels, perineural invasion, and peripheral bloodstream. The objective of this study was to assess the impact of SEMS placement on CRC lymph node metastasis.

**Methods:**

We retrospectively reviewed 48 patients with malignant colorectal obstruction treated with a temporary SEMS before elective surgery, and 51 patients with malignant colorectal obstruction who underwent elective surgery without prior SEMS placement.

**Results:**

No significant differences were found in the lymph node ratio (LNR) or in the results obtained from the logarithm of the ratio between positive and negative nodes (LODDS). Regarding recurrence, patients without SEMS had a fourfold higher risk of local recurrence compared to those with SEMS (19.6% vs. 6.3%), and a twofold higher risk of distant recurrence (31.4% vs. 14.6%). These differences were statistically significant for overall recurrence and for each local and distant recurrence individually (*P* = 0.02, *P* = 0.05, and *P* = 0.04, respectively).

**Conclusion:**

SEMS placement in obstructive CRC not only shows the potential to suppress tumor growth, but also reduce nodal spread, as no differences in LNR and LODDS values were observed when comparing preoperative SEMS placement in patients with advanced left CRC.

**Graphical Abstract:**

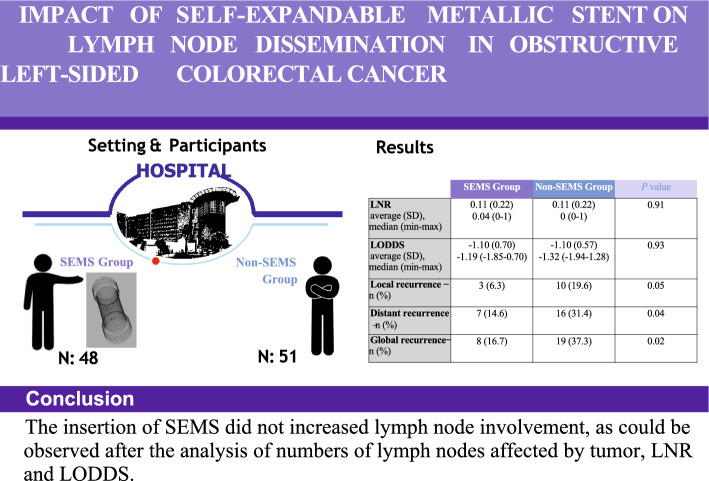

Colorectal cancer (CRC) is the fourth most common cancer and the second leading cause of cancer-related death worldwide [1]. Large bowel obstruction caused by advanced colon cancer occurs in 8% – 13% of patients with colon cancer [2–5]. The management of this serious clinical condition has been controversial [6]. Traditionally, obstructive CRC has been treated by emergency resection with anastomosis or Hartmann’s procedure [7, 8]. However, postoperative morbidities and mortality following surgical treatment of obstructive CRC are high [9].

Over the last decade, many articles have been published on the subject of self-expandable metallic stent (SEMS) for obstructive CRC. However, SEMS insertion in CRC has been associated with an increased risk of tumor perforation leading to peritoneal dissemination, as well as tumor cell spread through lymphatic vessels, perineural invasion, and peripheral bloodstream due to forced radial dilatation by SEMS placement [10–14]. Several studies, such as Sabbagh et al. [15], have reported a worse overall survival rate and a higher 5-year specific mortality in patients with left CRC previously treated with SEMS when compared to patients operated directly in the emergency department.

Optimal surgical removal of the tumor allows for adequate tumor staging, minimizing the risk of understaging for lymph node spread. The American Joint Committee on Cancer (AJCC) and the International Union Against Cancer (UICC) have recommended a minimum of 12 lymph nodes in pathologic CRC specimens for optimal tumor staging [16–19]. However, different studies have suggested that the lymph node ratio (LNR) should be considered a more reliable prognostic factor than the absolute number of nodes in stage III CRC [20–24]. On the other hand, the logarithm of the ratio between positive and negative nodes (LODDS) has also been proposed as a reliable variable in both 5-year overall survival and disease-free survival [25].

The main objective of the study is to evaluate the effect of SEMS placement on CRC lymph node metastasis. To this end, the absolute number of resected nodes, the number of positive nodes containing any number of malignant cells, the lymph node dissemination rate (LNR), and the logarithm of the ratio of positive and negative nodes (LODDS) were evaluated in comparison with patients undergoing CRC surgery without prior SEMS placement. The secondary endpoints were locoregional recurrence rate and the distant metastasis rate, as well as cancer-specific mortality, regardless of having previously undergone a SEMS placement.

## Materials and methods

**Fig. 1:**
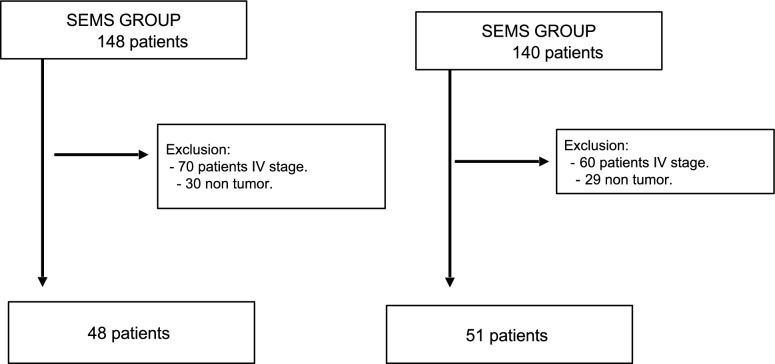
Study flow chart

This ambispective cohort study was conducted with patients who met the inclusion criteria and who had all been diagnosed with left obstructive CRC. The study took place between March 2008 and March 2019, and during this time, one group of patients in the cohort had a SEMS fitted as a bridge to a scheduled surgery, while the remaining patients did not undergo SEMS placement (Fig. 1).

For the group of patients who did have the SEMS places, the inclusion criteria were: a) diagnosis in the emergency room with left obstructive CRC; b) aged 18 or above; c) no signs of colonic tumor perforation; d) clinical stage II-III; e) no prior neoadjuvant chemoradiotherapy; f) active follow-up.

The inclusion criteria for the group of patients without SEMS were: a) patients diagnosed with left CRC undergoing scheduled surgery; b) over 18 years of age; c) tumor involvement of at least 3/4 of the circumference of the colon and with stenosing characteristics; d) absence of macroscopic perforation of the colon; e) clinical stage II-III; f) not having received neoadjuvant chemoradiotherapy treatment; f) complete follow-up after surgery.

In both groups, patients with obstructive CRC who underwent emergency surgery were excluded.

Emergency endoscopy was performed in all cases, using an Olympus flexible thin endoscope with a working channel. Once the stenosis was visualized, a 0.89 mm guidewire was introduced under fluoroscopic and endoscopic guidance. An uncovered self-expanding metallic stent was used. Technical success was considered when the stent was correctly placed, overcoming the stenosis, and stool evacuation was visualized through it. Clinical success was considered when there was complete resolution of obstructive symptoms. There were no complications related to the placement of the prosthesis. Patients who did not present technical or clinical success were excluded from the study.

The surgical intervention was performed by laparoscopic or open procedure following the principles of surgical oncology, with complete resection of the tumor macro and microscopically.

Patients in stages III and II with at least one high-risk feature, such as pathological T4 tumor, poorly differentiated or undifferentiated histology, presence of lymphovascular or perineural invasion, underwent adjuvant chemotherapy. Various chemotherapeutic regimens were employed, such as 5-fluorouracil and folinic acid (FUFOL), infusion of 5-fluorouracil, leucovorin, and oxaliplatin (FOLFOX), or weekly bolus of 5-fluorouracil, leucovorin, and oxaliplatin (FLOX).

### Pathology

#### Assessment of lymph node resection

Surgical specimens were fixed with 10% neutral buffered formalin. Traditional inspection and palpation methods were applied for lymph node resection. In cases where the number of nodes detected was less than 12, additional fat clearing with Carnoy's solution was performed to help better identify any hidden positive lymph nodes. Tissues were embedded in kerosene and histological analysis was performed using Hematoxylin–Eosin.

#### Assessment of Ki-67 immunohistochemical staining

Immunohistochemical (IHC) staining according to the avidin biotin procedure was performed using anti-Ki-67 (DAKO). In each analysis, CRC samples previously shown to stain with this antibody were used as positive controls. Phosphate-buffered saline was used as a negative control instead of the primary antibody.

For nuclear staining with Ki-67, the percentage of positive cells was calculated from at least 1,000 cancer cells from three randomly selected fields of vision using a high-power lens (× 400).

#### Assessment of intratumoral infiltration by lymphocytes (iTILs)

Tumor tissues were sectioned (5 µm) and prepared on polylysine coated slides and dried overnight at 37 °C for 24 h. The blocks were deparaffinized in xylene for 15 min and subsequently rinsed in ethanol. Endogenous peroxidase was blocked for 15 min in 10% hydrogen-peroxide methanol and subsequently the sections were rinsed in deionized water. Antigen retrieval for the markers consisted of boiling the tissue sections in 1 citrate buffered solution (pH 6) for 24 min in an 800 power microwave and then rinsed in Tris-buffered saline (TBS). The sections were then washed with phosphate-buffered saline (PBS) for 10 min and incubated overnight at room temperature with the following primary antibody: anti-CD8 + (mouse monoclonal anti-human CD8 antibody, clone C8/144B, code IS623; Dako, Glostrup, Hovedstaden, Denmark).

After overnight incubation with the markers, the sections were washed in TBS for 15 min and incubated with mouse Envision + visualization system (Dako) for 30 min. The sections were then washed in PBS for 15 min and developed in 3,3-diaminobenzidine tetrahydrochloride (DAB) with 0.002% hydrogen peroxide for 10 min, which resulted in a brown stain. Finally, the slides were rinsed in water, dehydrated in alcohol, and finally counterstained with hematoxylin.

The number of CD8 + iTILs was counted in each tumor using a microscope (Nikon, Tokyo, Japan). All sections from a single patient were first scanned at low-power magnification (× 10) and CD8 + TILs were distributed in the intratumoral region of the tumor.

Lymphocytes were quantitatively assessed as follows: with an optical microscope at × 400 magnifications, five high-power fields (HPF) were randomly selected, and the number of positive cells was counted to determine the number of lymphocytes per 250 µm2. The degree of infiltration was arbitrarily classified into three grades based on the number of infiltrating cells per unit area: poor, < 40; moderate, ≥ 40 but < 80; and severe, ≥ 80. For the statistical analysis, the moderate and severe groups were grouped together.

### Data collection

Data for this study was obtained a by retrospective peer review of electronic medical records and manual extraction of variables by two gastroenterological surgery specialists at our institution. Prospective data were also retrieved from the medical records once the study was initiated.

The clinicopathological variables collected were age, sex, preoperative physical condition of the patient according to the American Society of Anesthesiology (ASA), tumor location (descending colon, sigma), serum carcinoembryonic antigen level, type of surgery (left colectomy, sigmoidectomy), TNM 8th edition staging according to the American Joint Committee on Cancer (AJCC), degree of histological differentiation (well-differentiated, moderately differentiated, poorly differentiated and undifferentiated), whether the patient required adjuvant chemotherapy and the follow-up time.

The effect of SEMS placement was measured by: (1) number of resected nodes (RCC); (2) number of metastatic nodes; (3) ratio of positive lymph nodes containing malignant cells (LNR); (4) logarithm of the ratio of positive to negative nodes (LODDS); (5) Ki-67; (6) iTILs.

Locoregional recurrence and distant metastasis rates were determined by imaging and pathology reports; along with cancer-related deaths (> 30 days after treatment completion).

### Follow up

Maximum follow-up was 36 months, on average, patients were evaluated every 3 months during the first 2 years and every 6 months in the third year. Additional visits were made upon request. Follow-up was performed in general and digestive surgery outpatient clinics, as well as in medical oncology clinics, with complementary laboratory and imaging tests (carcinoembryonic antigen measurement analysis, thoracoabdominal CT and colonoscopy). Additional imaging studies were performed when local and/or distant recurrence were suspected.

All subjects gave informed consent prior to inclusion in the study. This study was reviewed and approved by the Research Ethics Committee of our hospital. The Approval No. was 2019_108.

### Statistical analysis

Student´s t-test was performed for continuous variables and Chi-square and Fisher´s exact test for categorical variables. Survival curves were generated using the Kaplan–Meir method and compared using a log-rank test. The exact proportion at 12, 24, and 36-month follow-up were calculated using the cumulative survival ratio and its asymptotic 95% confidence interval. Univariate and multivariate survival analyses were performed using the Cox proportional hazards regression model. The Hazard-Ratios (H-R) and its 95% confidence intervals were estimated. The assumption of constant H-R was verified. The statistical analysis was carried out using IBM SPSS version 22.0 (IBM Corp. Released 2013. IBM SPSS Statistics for Windows, Version 22.0. Armonk, NY: IBM Corp.). Comparisons of non-nested CoxPH models were carried out using R package version 0.0.0.9000 de Thomas Hielscher (2021). All two-sided p-value lower than 0.05 was considered statistically significant.

## Results

Of the 148 patients who underwent a SEMS, 48 were included and 100 were excluded because they did not meet the inclusion criteria of the study. The reasons for exclusion were 70 patients for presenting stage IV according to TNM staging at the time of diagnosis, 30 patients for diagnoses of non-tumor pathology in the pathology specimens after surgery.

Of the 140 patients who did not receive a SEMS, 51 patients were included and 89 patients were excluded. Sixty patients were excluded for being diagnosed as stage IV according to TNM before intervention, and 29 patients for diagnosis of non-tumor pathology in the pathology reports.

It was found 3 patients that were excluded due to not present technical success and 2 patients due to not present clinical success.

Table 1 shows the epidemiological characteristics of the sample, with no significant differences being found in these variables, nor in those related to the anatomopathological results of the tumor between patients with and without SEMS. However, there were differences in tumor location, distributed in a similar proportion between sigma and the descending colon in the non-SEMS group, respectively, and with a higher proportion of sigma in the SEMS (P = 0.02). The mean interval from SEMS placement to surgery was 12.5 days (CI95%: 10.8; 14.2).

**Table 1:** Epidemiological characteristics of the patients and anatomopathological findings of the tumor

	SEMS Group n = 48	non-SEMS group n = 51	*P* value
*Age* (years) mean (SD), median (min–max)	70.88 (11.5) 73 (45–88)	68.57 (10.33) 70 (44–94)	0.30
*Sex* (Female)—n (%)	26 (54.2)	29 (56,8)	*0.80*
*ASA*—n (%)			0.09
I-II	23 (47.9)	33 (64.7)	
III-IV	25 (52.1)	18 (35.3)	
Time from symptoms to surgery (days) average (SD), median (min–max)	13 (5.8) 13 (1–23)	76 (65.2) 50 (10–300)	< 0.001
*Location*—n (%)			0.02
Sigma	37 (77.1)	28(54.9)	
Descending	11 (22.9)	23 (45.1)	
*Histology type*—n (%)			0.08
Adenocarcinoma	45 (93.8)	42 (82.4)	
Mucinous Adenoc	3 (6.3)	9 (17.6)	
*Histology, Grade*—n (%)			0.31
Well-differentiated	12 (25)	20 (39.2)	
Moderately differentiated	21 (43.8)	17 (33.3)	
Poorly differentiated or undifferentiated	15 (31.3)	14 (27.5)	
*Stage*—n (%)			0.32
II	22 (45.8)	29 (56.9)	
III	26 (54.2)	22 (43.1)	
Adjuvant treatment	26 (54.2)	31 (60.8)	0.51
*Nº resected lymph node* average (SD), median (min–max)	19.06 (8.50) 17 (2–46)	19.5 (11.71) 17 (0–56)	0.85
*Nº positive lymph nodes* average (SD), median (min–max)	1.60 (2.47) 1 (0–14)	1.61 (3.65) 0 (0–23)	0.99
*LNR* average (SD), median (min–max)	0.11 (0.22) 0.04 (0–1)	0.11 (0.22) 0 (0–1)	0.91
*LODDS* average (SD), median (min–max)	−1.10 (0.70) −1.19 (−1.85–0.70)	−1.10 (0.57) −1.32 (−1.94–1.28)	0.93
Local recurrence—n (%)	3 (6.3)	10 (19.6)	0.05
Distant recurrence—n (%)	7 (14.6)	16 (31.4)	0.04
Global recurrence—n (%)	8 (16.7)	19 (37.3)	0.02
Mortality—n (%)	14 (29.2)	17 (33.3)	0.41

*DS* Standard deviation, *ASA* American Society of Anesthesiology, *LNR* lymph node ratio, *LODSS* Logarithm of the ratio between positive and negative nodes

Regarding regional and distant metastases, no significant differences were found between the groups in the number of resected nodes with a mean of 19.1 (8.5) in the group with SEMS and 19.5 (11.7) in the group without SEMS (P = 0.85), nor in LNR with a mean of 0.11 (0.22) in both groups (*P* = 0.9) nor for the results obtained for the variable LODDS with a mean of − 1.10 for both groups (*P* = 0.9).

**Fig. 2:**
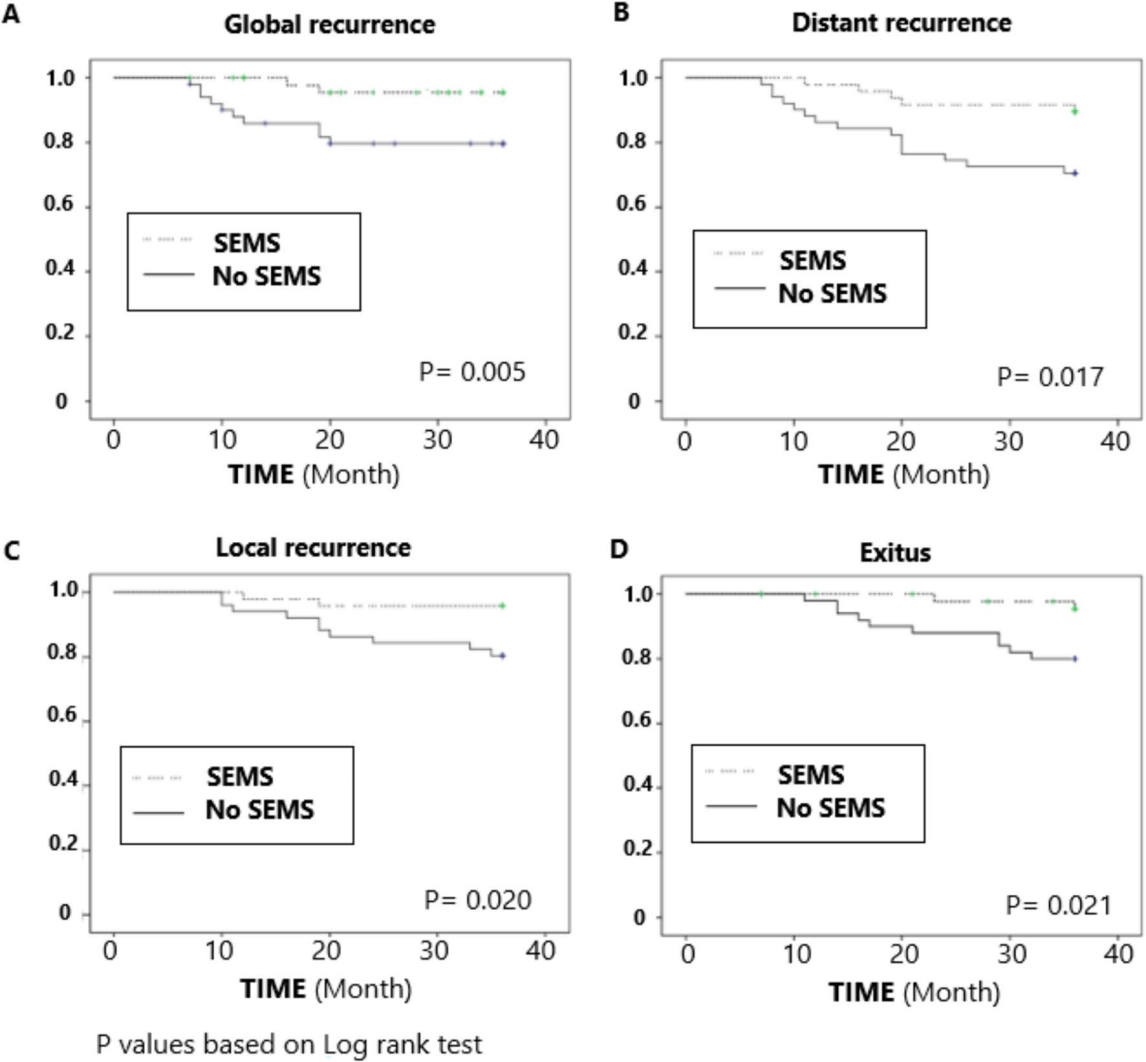
Kaplan–Meier survival curves. **A** Global recurrence, **B** Distant recurrence, **C** Local recurrence, and **D** Death

Figure 2 shows Kaplan–Meier analysis for global recurrence (a), distant recurrence (b), local recurrence (c), and mortality according to self-expanding metallic stent. SEMS group showed lower global (*p* = 0.005), distant (*p* = 0.017) and local (0.02) recurrence and lower mortality (0.021) than no SEMS group. Table 2 shows that during follow-up the percentage of patients without recurrence in the *SEMS* group was 95.7% at 12 months and 89.2% at 36 months compared to 84% and 64% in the group without SEMS, respectively. Thus, at 12 months, patients without SEMS almost quadrupled the overall recurrence risk (risk of 16%) of patients with SEMS (risk of 4.3%), a proportion that was maintained until the end of follow-up at 36 months. Regarding local recurrence, the risk of patients without SEMS quadrupled that of patients with SEMS (19.6% vs 6.3%) and in distant recurrence almost tripled (31.4% vs 14.6%), with statistically significant differences between the groups considering global recurrence and, separately, each local and distant recurrence (*P *= 0.005, *P* = 0.017, and *P* = 0.020, respectively). Table 3 shows the incidence ratio of global recurrence adjusted for age, sex, and LNR (model 3), being 4 times higher in patients without prosthesis when compared to patients with prosthesis (HR- = 4.4; CI95%: 1.6; 12.3). Similar to the result obtained when adjusting for LODDS instead of LNR, model 4 was not significantly different in goodness-of-fit to model 3 (P = 0.08).

**Table 2:** Survival analysis with Kaplan–Meier

	Patients with no events, % (95%CI)						Test Log- Rank	
	12 months		24 months		36 months		Chi-square	P-value
	Prosthesis	No Prosthesis	Prosthesis	No Prosthesis	Prosthesis	No Prosthesis		
Global recurrence	95.7 (90.0–100)	84 (73.8- 94.1)	89.2 (80.1- 98.2)	70 (57.2- 82.7)	89.2 (80.1- 98.2)	64 (51.7- 77.3)	7.84	0.005
Local recurrence	97.9 (94.8- 100)	94.1 (87.6- 100)	95.8 (90.1- 101.5)	84.3 (74.3- 94.3)	95.8 (90.1- 101.5)	80.4 (69.4- 91.4)	5.42	0.020
Distant recurrence	97.9 (93.8- 100)	86.3 (76,9- 95.7)	91.7 (83.9- 99.5)	74.5 (62.5- 86.5)	89.6 (81.0- 98.2)	70.6 (58,1- 83.1)	5.69	0.017
Mortality	100 –	98 (94.1- 100)	97,8 (93.4- 100)	88 (79.0- 97.0)	95,4 (89.3- 100)	80 (68.8- 91.1)	5.29	0.021

Decreased Ki-67 expression was observed in surgically resected specimens in the SEMS group compared to the non-SEMS group (15.76 ± 21.67 vs 11.92 ± 14.58; *P* = 0.97). On the other hand, a higher frequency of intratumoral infiltration by lymphocytes in the SEMS group compared with the non-SEMS group (29% vs 23.3%; *P* = 0.58).

Mortality, in the non-SEMS group was 20% at 36 months versus patients with SEMS with a risk of 4.6% (see Table 3). The difference between the survival curves for patients with and without SEMS was significant (*P* = 0.021) and is shown in Fig. 2. The crude H-R for mortality was for patients without SEMS (model 1) was 5.0 (CI95%: 1.1; 22.7) and adjusted for age and LNR (model 2) was 6.90 (CI95%: 1.4; 33.05) (see Table 4). If we adjust for LODDS (model 3), the H-R of patients without SEMS is reduced to 5.89 (CI95%: 1.24; 27.91), although model 3 was not significantly different in goodness-of-fit to model 2 (*P* = 0.25).

**Table 3:** Cox regression analysis for overall recurrence

	Overall recurrence n(%)		Model 1	Model 2	Model 3	Model 4
	Yes	No	Wald H-R (95% CI) p-value	Wald H-R (95% CI) p-value	Wald H-R (95% CI) p-value	Wald H-R (95% CI) p-value
No Prosthesis	18 (35.3)	33 (64.7)	6.72 3.71 (1.38–9.99) 0.010	8.36 4.52 (1.63–12.59) 0.004	8.14 4.43 (1.60–12.30) 0.004	7.86 4.26 (1.55–11.72) 0.005
Prosthesis (ref)	5 (10.4)	43 (89.6)				
Age				3.19 1.04 (0.99–1.10) 0.07	2.44 1.04 (0.99–1.09) 0.12	1.35 1 (0.98.1.10) 0.24
Male				2.29 1.25 (0.55–2.85) 0.59	0.78 1.47 (0.62–3.48) 0.37	1.34 1.67 (0.7–4) 0.25
LNR					3.79 4.4 (0.99–19.45) 0.05	
LODDS						9.40 2.40 (1.37–4.21) 0.002

*LNR* Lymph node ratio, *LODDS* Logarithm of the ratio between positive and negative nodes, Hazard-Ratio (H-R)

**Table 4:** (a) Contingency table that includes mortality according to prosthesis status (prothesis/non prosthesis, (b) Simple and (c) and (d) multiple Cox regression analyses using mortality as event and time to event as dependent variable

Variables	(a) Mortality—n(%)		(b) Model 1	(c) Model 2	(d) Model 3
	Yes	No	Wald H-R (95% CI) p-value	Wald H-R (95% CI) p-value	Wald H-R (95% CI) p-value
No Prosthesis	10 (19.6)	41 (80.4)	4.28 5 (1.10–22.66) 0.04	5.86 6.91 (1.46–33.05) 0.02	4.98 5.89 (1.24–27.91) 0.03
Prosthesis (ref. category)	2 (4.2)	46 (95.8)			
Age (years)				5.0 1.09 (1.01–1.17) 0.05	3.60 1.10 (0.99–1.14) 0.06
LNR				3.79 5.22 (0.99–27.52) 0.05	
LODDS					6.46 2.32 (1.21–4.44) 0.01

*LNR* Lymph node ratio, *LODDS*: logarithm of the ratio between positive and negative nodes, Hazard-Ratio (H-R)

## Discussion

The study of lymph nodes in the operative specimen of patients operated on for CRC is important for establishing the prognosis and staging of the patient. However, the minimum number of resected lymph nodes is not achieved in all resected CRC specimens due to multiple factors [26, 27]. To overcome this limitation, different publications have indicated that LNR is a better prognostic indicator and an alternative staging method in patients with CRC [28–30].

LNR is defined as the ratio between the number of positive nodes and the total number of nodes removed. Currently, LNR is considered more reliable as a prognostic tool in stage III CRC [20, 21, 23, 31, 32] than the absolute number of lymph nodes containing malignant cells, and a more useful prognostic marker than the number of metastatic nodes [33]. These results have also been demonstrated in patients with pancreatic, breast, and gastric cancer [34–36]. Numerous studies have shown that patients with low LNR have a similar overall survival to node-negative patients [37]. The apparent success of LNR as a better prognostic factor, as opposed to simple metastatic lymph node counting, may lie in the fact that it adds a new dimension in staging, demonstrating not only the aggressivity of the tumor, but also of the patient's immune response.

Recently, the log odds of positive nodes (LODDS) of different tumors have also been proposed as a reliable variable in both 5-year overall survival and disease-free survival. This variable is defined as the logarithm of the ratio between positive and negative nodes [25]. This variable has been evaluated in gastric cancer, breast cancer and CRC, and has a greater predictive value on survival than the absolute number of affected nodes per tumor and the TNM [38–40].

It has been suggested that forced radial dilatation by SEMS placement in the management of left CRC would not only increase the risk of perforation but would also lead to dissemination of tumor cells within the lymphatic vessels, perineural invasion, and peripheral bloodstream [13, 41–43]. In our study, we have analyzed the involvement of the lymph nodes by tumor cells after the placement of SEMS in CRC. Both the mean number of resected lymph nodes and the number of positive nodes were similar in both groups. Moreover, we observed no differences in either LNR or LODDS between the group of CRC patients initially treated with SEMS and the control group (Table 1). The reasons for this fact remain to be determined. A possible explanation for the results obtained in our study would be the mechanical effect caused by SEMS, by generating competition for the space between the tumor and the stroma. In this regard, in a study by Matsuda et al. in 25 patients with obstructed left-sided CRC treated with SEMS, it was established that the mechanical compression exerted by SEMS on obstructive CRC could suppress tumor cell proliferation. In this regard, mechanical manipulation of the CRC after SEMS placement resulted in a decrease in Ki-67 expression [43].

Ki-67 antigen is expressed by cells in the G1-, G2-, S-, and M-phase of the cell cycle and is commonly used as a marker of proliferative activity [44]. High Ki-67 expression is significantly correlated with poor overall survival and disease-free survival [45]. The tumor microenvironment plays an important role in the prognosis of colorectal cancers [46]. In recent years, numerous studies have demonstrated the prognostic significance of the colorectal cancer-associated lymphocytic response [47]. The presence of iTILs has been associated with a better prognosis in colorectal cancer [48–54].

After analyzing Ki-67 in our study and the immune response evaluated by intratumoral infiltration, it was observed that although there were no significant differences, a lower Ki-67 was observed in the SEMS group vs the non-SEMS group, 15.76 ± 21.67 vs 11.92 ± 14.58 (p = 0.97), as well as a higher frequency of intratumoral infiltration by lymphocytes in the SEMS group. Thus, the percentage of moderate-high intratumoral infiltration was 29% in the SEMS group and 23.3% in the non-SEMS group (p = 0.58). The non-observation of significant differences may be related to the power of the study due to the small sample size of the study.

The communication pathways between the tumor and its microenvironment are multiple but can be classified into biochemical and mechanical signals. Such biochemical signals have been extensively studied in previous publications [55, 56], however, only more recently are mechanical signals being studied. In vitro studies on tumor models have revealed that compression forces applied to the tumors lead to a drastic reduction in the proliferation rate of tumor cells without an increase in cell apoptosis [57, 58]. Delarue et al. [59], described that compressive stress in vitro studies inhibited colonic tumor cell proliferation by blocking the G1 phase of the cell cycle through overexpression of the cyclic cyclin-dependent kinase inhibitor p27kip1. Reduced expression of p27kip1 is prevalent in a wide variety of tumors, including CRC, and its decreased expression is also correlated with more advanced tumor stages and worse outcomes depending on distinct types of cancer [60, 61].

On the other hand, different studies have evaluated the histocytology in several CRC pieces with SEMS, observing inflammatory changes similar to inflammatory bowel disease [62–64]. Galon et al*.* [65] have described that the type, density and location of immune system cells in CRC could predict patient survival more reliably than standard pathology assessment. In different studies, it has been observed that patients with prominent lymphocytic infiltration in the primary tumor had better survival [66, 67].

Finally, other factors may influence the prognosis of CRC. The short-term outcome of SEMS placement is known to result in lower rates of suture failure and stoma creation. Obstructive colitis (OC) present in obstructive CRC has been identified as a risk factor for anastomotic leakage during CRC resection [68]. In addition, Mekata suggested that tumor may spread to mucosal areas damaged by OC and favor intraluminal growth [69]. However, further studies are needed to clarify this idea.

Regarding the limitations of the study, we must acknowledge that since it is an ambispective cohort, it includes the limitations inherent to such studies. Firstly, this study is based on a cohort from 2008 to 2019, so the decision of SEMS in the management of left CRC depended on the individual criteria of the surgeon. Currently, the performance of colonoscopy prior to surgery has been protocolized in our service, being performed in all patients who undergo the placement of SEMS in the management of left CRC. Although there were no significant differences in the histology of the specimen, we acknowledge that in the non-SEMS group, there was a higher percentage of mucinous adenocarcinoma (17.6% vs 6.3%). This fact should be taken into account since mucinous adenocarcinoma of CRC are related to less responsive to neoadjuvant and adjuvant chemotherapy as compared to those with non-mucinous colorectal adenocarcinoma, due to the mucinous colorectal adenocarcinoma histology [70–72]. Furthermore, it has been described that mucinous colorectal adenocarcinoma patients had lower OS rates when they received the same therapies as non-mucinous colorectal adenocarcinoma patients [73]. On the other hand, another of the study's biases is that stage III patients received different types and number of chemotherapy treatments. Therefore, we believe that further studies are needed to clarify the effect of SEMS on the development of lymph node dissemination and recurrence as well as survival of CRC.

In conclusion, and according to the results of our studies, the insertion of SEMS did not increase lymph node involvement, as could be observed after the analysis of numbers of lymph nodes affected by tumor, LNR, and LODDS. Moreover, in our studies we were able to observe a lower Ki-67 and a greater intratumoral lymphocyte infiltrate in the SEMS group than in the non-SEMS group. We believe that the insertion of SEMS could suppress the proliferation of tumor cells and stimulate the immune system, thus reducing the spread of the tumor to the lymph nodes.
